# Validation of the Global Scale for Early Development (GSED) for children 0 to 36 months of age in the Pacific: protocol

**DOI:** 10.1016/j.puhip.2025.100615

**Published:** 2025-05-11

**Authors:** S. Howells, B. Lam, D. Kakiakia, B. Temakei Tebano, R. Tekeraoi, R. Katokita, S.A. Brinkman

**Affiliations:** a Education Futures, University of South Australia, North Terrace, Adelaide, 5000, South Australia, Australia; b Ministry of Education, Government of the Republic of Kiribati, South Tarawa, Kiribati

**Keywords:** Child development, Validity, Reliability, Epidemiology, Global health

## Abstract

**Objectives:**

Early childhood starting at conception is a period of rapid development and has implications for health and well-being throughout the life-course. Validated measures are critical for countries interested in population level monitoring of child development and to evaluate policies and services aimed at enhancing children's health and development. This project aims to address this need for children aged 0–36 months in Kiribati, a small and widely dispersed island nation in the central Pacific Ocean, by adapting and validating the Global Scale for Early Development Short Form (GSED SF). This study contributes uniquely to the literature as it is the first time that the GSED has been adapted and applied in the Oceania-Pacific region.

**Study design:**

Psychometric validation study of the GSED SF in Kiribati.

**Methods:**

The Global Scale for Early Development Short Form (GSED SF) adaptation and validation study will involve 500 children, 100 each from five randomly selected villages in South Tarawa, Kiribati. Validity testing will involve established steps: face validity, cultural and context neutrality, test-retest reliability, inter-rater reliability, construct validity and discriminant validity. We will evaluate measurement invariance including differential item functioning and differential test functioning to ensure that the GSED SF is fair and unbiased.

**Conclusions:**

This project will provide Kiribati with a tool for monitoring and evaluation of early child development in children from birth to 36 months at the national and programmatic level. The study will also provide the first validation of the GSED SF in the Pacific region.

**What this study adds**.

• The WHO GSED has yet to be implemented in the Pacific region;
• contributes to the international evidence behind the applicability of the GSED to different cultures and contexts;
• provides local evidence for its reliability and validity for Small Island States in the Pacific.

**How this study might affect research, practice or policy**.

• This study will provide the first validation of a tool to assess early child development (ECD) for children under 3 years of age for the Pacific region. This tool affords government, donors, non-government organisations, and communities with a validated instrument to conduct future evaluations of the impact and effectiveness of programs, interventions, and services on child development.
• The instrument will also allow identification of populations most in need of support, tracking ECD trajectories over time at a population level, and monitoring of the benefits of national-level policies

## 1 Introduction

Early childhood, beginning at conception, is a critical time for rapid brain and overall development, significantly influencing health and well-being throughout an individual's life. Optimal child development is fostered in environments that are biologically, socially, emotionally, and cognitively nurturing [1]. By contrast, suboptimal development can impact long-term developmental potential and outcomes including poorer educational and employment attainment, and lower quality of life [2,3].

This crucial phase in a child's life is acknowledged in the United Nations Sustainable Development Goal (SDG) 4.2, which seeks to monitor both access to quality services and the measurement of children's developmental outcomes across the world [4]. While it is essential to monitor early child development (ECD) to identify where greater supports are needed, historically there has been a gap in internationally validated measures, especially for children prior to three years of age.

However, there are recognized challenges regarding the universal applicability of developmental domains and measurements of ECD across cultures and contexts [1,[5], [6], [7]], with growing recognition of the importance of ECD frameworks that explore specificity [8], whilst also allowing cross cultural and longitudinal monitoring [7]. The recent development and ongoing validation of the Global Scale for Early Development (GSED) by the World Health Organization (WHO) has responded to this challenge with a population measure that aims to generate internationally comparable data for children under three years of age [9].

To date, validation studies of the GSED have been conducted across numerous low and middle-income countries (LMIC), although none in the Oceania Pacific region. Initial WHO validation involved two rounds: the first in Bangladesh, Pakistan, and Tanzania [10]; with a subsequent round being undertaken in Brazil, Côte d’Ivoire, the Netherlands and China [9,11]. A further recent validation study has been conducted in Kenya (GSED Long Form (GSED LF)) [12], while a feasibility and implementation study has been performed for the GSED LF and GSED Short Form (GSED SF) in Ethiopia [13]. Kiribati, a small and widely dispersed island nation in the central Pacific Ocean has already seen success in adapting and implementing the early Human Capability Index (eHCI) [[14], [15], [16], [17], [18]] which is a population level measure of early child development validated for use with children three to six years of age. The integration of the recently developed GSED SF with the eHCI would be particularly advantageous, allowing for a seamless and continuous monitoring framework to track ECD from birth to six years, ensuring that Kiribati has a comprehensive approach to ECD assessment.

This project aims to adapt and validate the GSED SF including the testing of measurement invariance and psychometric properties, for use in Kiribati. The project will initially determine the instrument's face validity and cultural and context neutrality, followed by test-retest reliability, inter-rater reliability at the score and item level, and construct validity. The GSED offers potential to enable the Kiribati government and community agencies to effectively monitor and evaluate ECD with an instrument that allows for international comparison for ages 0–36 months.

### 1.1 The GSED

In 2023, the WHO launched the GSED, a tool designed to assess ECD up to 36 months of age in a variety of cultural contexts [9,10,19]. This instrument was crafted to support the evaluation of ECD both at the population and programmatic level. The GSED package v1.0 was released in February 2023, and it is this material that will be used for validation in Kiribati.

The GSED is an open-access tool, characterized by its standardization, cultural neutrality, ease of administration, and comprehensibility for caregivers [20]. The GSED consists of two measures, a caregiver-reported Short Form (GSED SF) (137 items) and a directly administered Long Form (GSED LF) (155 items distributed evenly across 3 streams) [10]. The SF and LF measure overall development across the cognitive, motor, language and socioemotional domains appropriate to children under three years of age. Responses allow for calculation of a single score for each child, the D-score, which is a holistic measure of ECD. The SF and LF are psychometrically aligned and produce metrics on the same age-ordinal scale and quantify the same latent constructs.

Initial validation scores for the GSED, based on data from Pakistan, Tanzania and Bangladesh, demonstrated high inter-rater and intra-rater reliability and intraclass correlation coefficients (>0.98 across all forms) [21,22]. Furthermore, high correlations were also demonstrated with Bayley III (*r* > 0.88 for all domains). Recent data from Kenya for the GSED LF however demonstrated weaker results overall for concurrent validity correlations with the Bayley- III and CREDI. In the latter study, scores were positively associated with cognitive, language, and motor domains of the Bayley-III (*r* = 0.22–0.30), with the strongest associations between D-scores and Bayley-III fine motor and gross motor development (*r* = 0.27 and *r* = 0.30, respectively). By contrast, no significant association was detected between the GSED D-score and the Bayley-III socioemotional subscale. Due to the pragmatics of data collection in Kiribati, this study will be limited to adapting and validating the GSED SF.

### 1.2 Child development in Kiribati

Kiribati is home to around 120,000 people, inhabiting 23 islands scattered across 3.5 million square kilometres in the central Pacific. Forty percent of its population are under 18 years of age. As of 2016, Kiribati was ranked 137th in the Human Development Indicators by the United Nations Development Programme, reflecting its development challenges.

Kiribati is among the first countries in the world to have undertaken a national census focused on the health and development of young children. In 2017, a census of all children aged 3- to 5-years highlighted a range of ECD challenges. The census utilised the locally adapted early Human Capability Index (eHCI), and key findings included limited healthcare access, significant undernutrition (approximately one-third of the children showed stunting), variations in preschool attendance rates, and disparities in ECD outcomes across different islands[18]. It also identified areas for enhancement in caregiver-child interactions, which are crucial for children's development. These insights provided a crucial foundation for the launch in 2023 of the inaugural Kiribati Early Childhood Development Policy (KECD Policy). This underscores the country's dedication to upholding children's rights and emphasizes the importance of monitoring child development across the nation. There is, however, a gap in understanding the development of children under the age of three years, with information limited to basic mortality and morbidity data.

## 2 Methods

### 2.1 Study Design and study sites

The GSED SF validation study will use a cross-sectional design. Study sites include selected villages across South Tarawa, the urban capital of Kiribati, and consequently the sample will not be nationally representative. Both primary and secondary caregivers of children will be recruited.

#### 2.1.1 Preparation and feasibility

The instrument was translated in 2024 following the procedures specified by the WHO [23], with significant input and feedback provided by the Ministry of Education (MOE), Government of Kiribati, and WHO representatives. The WHO GSED SF User Manual and Item Guide were also translated for fieldworkers. Stakeholder engagement and adaptation of the instrument to the local context, including translation into the Te-Kiribati language, allows local ownership of the process, results, and dissemination, to promote future policy, advocacy, and monitoring, as well as national commitments.

Face validity and cultural and context neutrality of the GSED 10.13039/100013751SF plus additional study measures and supporting materials will be conducted through purposive sampling of diverse stakeholders including senior staff from government ministries including Ministry of Education, 10.13039/100009647Ministry of Health and Medical Sciences (MHMS), non-government stakeholders, caregivers, and senior community members. Face validity will involve the subjective assessment of the presentation and relevance of the survey to test the survey items in terms of the clarity, lack of ambiguity, ability to translate into Te-Kiribati, relevance, and reasonableness. Cultural and context neutrality testing of GSED SF items ensures that items are not biased against the cultural practices and expectations of children in Kiribati. Whilst GSED was designed to be neutral and universally valid across cultures and contexts, it is recognized that adaptations may be warranted for specific items across different settings [23].

### 2.2 Study sample

The study sample will include caregivers of children between 0 and 36 months of age, living in the study area. Inclusion criteria include that the caregiver has a child aged between 0 and 36 months, is available to participate, and is aged 16 years or over.

#### 2.2.1 Recruitment and consent

**Fig. 1:**
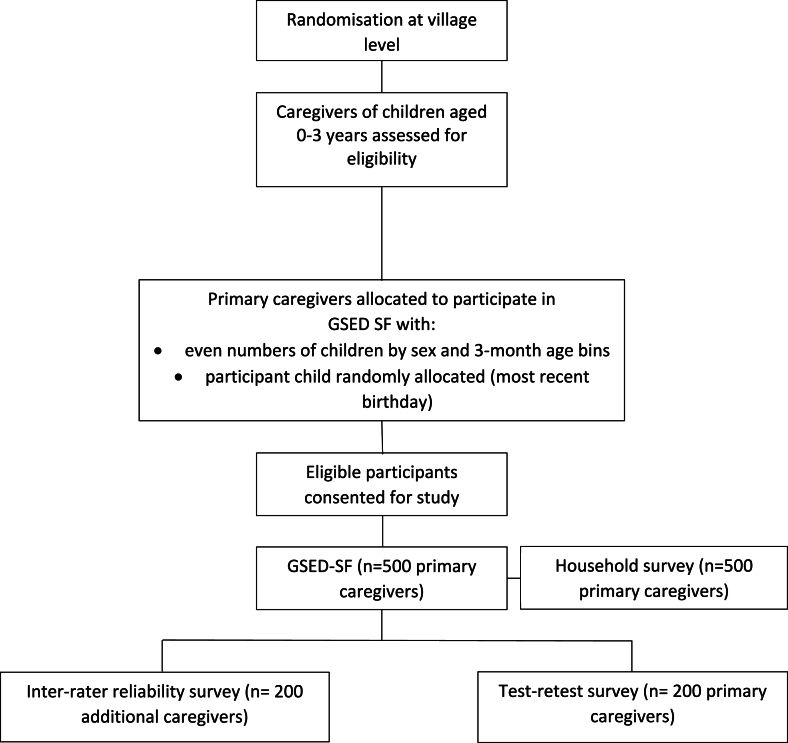
Participant flow diagram.

We will randomly select five villages and for each we will recruit a sample of convenience of 100 families. We will seek endorsement from the local councils/maneaba of South Tarawa to approach families within their area to invite them to participate. To minimise clustering of scores within households, if more than one child per caregiver unit (including siblings, twins) meets the inclusion criteria, data will be collected on the child with the most recent birthday (Fig. 1). The primary caregiver will be defined as the person who provides most of the care for the child and thus has the greatest knowledge of the child's abilities and behaviours, while the additional caregiver will comprise a person who provides care and has knowledge of the child. Caregivers will be provided with information about the project, participation requirements, and details of the management of personal information and data, and will be asked to provide consent for their participation in the project. Refusal to participate and dropouts will be recorded and replaced.

#### 2.2.2 Sampling scheme

The study sample for recruitment is caregivers of 500 randomly selected children between 0 and 36 months of age. Five hundred primary caregivers will complete the GSED for one of their children. Consistent with the World Health Organization's protocol for validation, the sample will consist of even numbers of children by sex and 3-month age bins. Inter-rater reliability will be tested for the first 200 of the 500 children, whereby two caregivers will simultaneously but separately respond to the GSED SF for the same child. Test-retest (repeat-test reliability) will involve the last 200 caregivers from the original sample, re-answering the GSED SF for the same child, one to two weeks later, by the same fieldworker.

### 2.3 Data collection

#### 2.3.1 Measures

##### 2.3.1.1 GSED SF

The development of the GSED has been previously described by McCray, McCoy [19], with detailed background and implementation information provided within the WHO Technical Manual [10]. The GSED SF is caregiver reported and intended to capture ECD at the population level or for program evaluation. It is based on 22 established instruments using cross-sectional and longitudinal data from over 70,000 children from 31 countries, and measures cognitive, motor, language, and socio-emotional domains. All items have yes/no/don't know answers. The survey comprises 139 items and applies an adaptive testing approach in which items are presented at the ability level of the respondent, with subsequent items dictated by the ability of the child, thus reducing the number of questions required per respondent. The GSED SF is available in both paper- and app-based (GSED App) versions. This study will use the latter. Annexed materials such as visual aids are available to provide visual cues to the caregiver to assist in question understanding.

##### 2.3.1.2 Contextual and demographic measures

A paired household survey will provide contextual information including family and household structure, socioeconomic details, access to and use of health and education services, and community context. The household survey will be developed by the study team and will also include items regarding caregiver practices and involvement. The items will be developed based on previous Government of Kiribati censuses, common relevant survey scales as used in the Kiribati Demographic and Health Survey and UNICEF MICS instruments, and previous household/caregiver questionnaires developed by the Principal Investigator (SB) for use in LMICs.

##### 2.3.1.3 Schedule

Data collection is scheduled for September to December 2024 over one to three visits and will be conducted in the caregiver's home or at a site convenient to the caregiver and fieldworkers to ensure privacy and safety of participants and fieldworkers.

### 2.4 Training and quality control

#### 2.4.1 Fieldworker recruitment

Fieldworkers will be recruited from local communities and where possible we will aim to recruit individuals who possess a secondary school qualification and some experience of working with families.

#### 2.4.2 Fieldworker training

Following the WHO GSED guidelines, fieldworkers will be provided with the official GSED SF user manual (Global Scales for Early Development v1.0 Short Form (caregiver-reported) User Manual) [24] which provides guidance for fieldworkers on the background and administration of the SF. The manual includes information on the background to the GSED SF, guidelines on the correct administration of the form/items, and advice on dealing with unexpected and challenging situations. The manual will be used in conjunction with the GSED SF Item Guide [25] which provides detailed information on the administration of each item.

An initial GSED SF training workshop in conjunction with an online training module will be conducted by WHO staff and undertaken by senior study staff including the in-country Research Coordinators. This will be followed by a 5-day training and certification workshop (conducted by a trained local master trainer) to skill local fieldworkers on the accurate administration of the GSED SF. The latter workshop will be conducted in English and Te-Kiribati and supported by materials adapted and translated into Te-Kiribati, including the GSED 10.13039/100013751SF User Manual and Item Guide, slide presentations, discussion, audio-visual aids, and practice exercises.

Following the workshop, fieldworkers will have access to ongoing in person support from the in-country Research Coordinators, in addition to phone and web-based support by the project team.

### 2.5 Sample size

The GSED validation is not intended to yield nationally representative results. Instead, we will use a convenience sample of 500 children and report 95 % confidence intervals for all estimated validity metrics. The sample will additionally be able to estimate the D-score distribution for Kiribati matched against the original applications of the GSED in the pilot countries. Inter-rater and test-retest reliability will be assessed by sub-samples of 200 children respectively.

### 2.6 Statistical analyses

We will undertake analyses to determine the GSED SF's measurement properties to understand its reliability in assessing ECD in Kiribati (Table 1).

**Table 1:** Proposed psychometric analyses.

Construct	Analysis method	Sample size	Comparator tools	Planned sub-group analyses
Face validity	N/A	20	N/A	N/A
Cultural and context neutrality	N/A	20	N/A	N/A
Test-retest reliability	ICC (score level) Gwet AC1 (item level) Bland-Altman analysis	200	Expected ICC of above 0.9 Gwet AC1 above 0.4	Stratified by gender
inter-rater reliability	ICC (score level) Gwet AC1 (item level) Bland-Altman analysis	200	ICC above 0.8 Gwet AC1 above 0.4	
Construct validity	Rasch	500	Infit and outfit statistics using Mean Square (distance from 1) and Z-standardised values (distance from 0) DIF by logistic regression method	DIF by gender, caregiver education, socioeconomic status
Discriminant validity	Descriptive known-groups comparison	500	Group comparisons using maternal education, home learning opportunities, home environment, socioeconomic status	Scores compared between high and low categories

Rasch models will be fitted to the item data. This statistical approach ensures the developmental milestones assessed are age-ordinal, reflecting the expected progression of skills with age and will facilitate the creation of a Developmental Score (D-score), providing a single continuous metric indicative of a child's developmental level. Measurement invariance tests will then be conducted investigating differential item functioning and differential test functioning to ensure that the GSED SF is fair and unbiased including across gender, caregiver education, and socioeconomic status. Our results will be compared to previous validation studies using the GSED in different populations.

Intraclass Correlation Coefficient (ICC 2,1) will be used to explore reliability at the D-score level with Gwet AC1 applied at the item level. Both test-retest and inter-rater reliability will be assessed through Intraclass Correlation Coefficient analyses along with visualisation through Bland and Altman plots to explore discrepancies between raters [26]. Discriminant validity will be assessed by determining if the GSED discriminates by gender, home stimulation and socioeconomic indicators including maternal education level.

The study findings will be distributed widely to a diversity of stakeholders at the local, national, and international level following a comprehensive culturally appropriate dissemination strategy.

## 3 Discussion

In Kiribati, there is currently no established practice for assessing ECD under three years of age. The validation of the GSED SF in Kiribati will provide a tool which will enable the measurement and monitoring of developmental progress at the population level and permit evaluation of the effectiveness of national policies. When used in conjunction with the eHCI, validated ECD assessment from birth to 6 years of age will be possible. Adding a child development measure to the current health indicators allows for a focus on thriving and not just surviving.

A limitation of our study is the logistical challenge of reaching the widely dispersed island communities of Kiribati. Consequently, this study will be confined to the villages of South Tarawa. Representativeness is not required for instrument validation studies however it is important for the sample to cover the developmental range expected in Kiribati. The sample coverage and size are expected to be robust enough to provide a reliable assessment of the tool's validity.

This study is being conducted in close collaboration with the Government of Kiribati, aiming to build local capacity of government staff and local fieldworkers in the independent and effective use of the GSED SF. Importantly, the tool will also permit future community level child assessment and program evaluation aligned with the key objectives of the KECD Policy.

From an international perspective, this research will add to the literature investigating the performance of the GSED SF in a new context and culture. The GSED has not formerly been applied in Oceania and as a relatively new instrument intended to support countries to measure their progress against Sustainable Development Goal 4.2, such validation is important. Further, this research will contribute vital data from the Pacific to the growing international comparative data collated by WHO.

## Statement of ethical approval

Although we have formal research approval from the Government of Kiribati, there is no ethics process in Kiribati, therefore an application has been sought and approved by the Human Research Ethics Committee at the University of South Australia (UniSA HREC) (ID205559). The study will comply with the National Statement on Ethical Conductin Human Research (https://www.nhmrc.gov.au/about-us/publications/national-statement-ethical-conduct-human-research-2023) and the Australian Code for the Responsible Conduct of Research https://www.nhmrc.gov.au/about-us/publications/australian-code-responsible-conduct-research-2018). Research data is re-identifiable to allow for possible future data linkages. Separation principals will be applied where identifiable aspects of the data will be kept separate from non-identifiable research data. Only the team will have access to the identifiable information for re-identification, and data will be stored securely in accordance with UniSA policies for a period of five years post publications. Informed consent will be obtained from all survey respondents. We will prepare and share a report and disseminate findings with stakeholders through workshops in addition to presenting findings at local and international conferences and through publication in peer-reviewed journals.

## Ethics and dissemination

The protocol was approved by the Human Research Ethics Committee at the University of South Australia (UniSA HREC) (ID205559) Findings will be disseminated to all stakeholders through presentations and synthesised reports.

## Authors' contributions

SB and BL developed the methodology. SH wrote the original manuscript and SB, RT, RK, DK, BT and BL provided feedback and revisions on the draft manuscript. All authors approved the final version.

## Data statement

Re-identifiable data will be stored in a secure server at The University of South Australia, and according to the UniSA Research Storage principles. The datasets generated and/or analysed during the current study are not publicly available to protect participant privacy, however de-identified data are available from Professor Brinkman on reasonable request.

## Funding statement

This work was supported by funding from Foreign, Commonwealth Development Office, UK, and 10.13039/501100008631New Zealand Ministry of Foreign Affairs and Trade.

## Declaration of competing interest

The authors declare that they have no known competing financial interests or personal relationships that could have appeared to influence the work reported in this paper.

## Figures and Tables

**Table:** 

CREDI	Caregiver reported early development Instrument
ECD	Early Child Development
eHCI	Early Human Capability Index
GSED	Global Scale for Early Development
KECD Policy	Kiribati Early Childhood Development Policy
LF	Long form
MICS	Multiple Indicator Cluster Survey
MOE	Ministry of Education
MHMS	Ministry of Health and Medical Services
SF	Short Form
WHO	World Health Organization
